# Cardiac Magnetic Resonance Predicting Outcomes Among Patients at Risk for Cardiac AL Amyloidosis

**DOI:** 10.3389/fcvm.2021.626414

**Published:** 2021-06-29

**Authors:** Ali M. Agha, Nicolas Palaskas, Amit R. Patel, Jeanne DeCara, Purvi Parwani, Cezar Iliescu, Jean B. Durand, Peter Kim, Saamir Hassan, Gregory Gladish, Hans C. Lee, Gregory P. Kaufman, Juan C. Lopez-Mattei

**Affiliations:** ^1^Department of Medicine and Center for Cardiometabolic Disease Prevention, Baylor College of Medicine, Houston, TX, United States; ^2^Division of Internal Medicine, Department Cardiology, University of Texas MD Anderson Cancer Center, Houston, TX, United States; ^3^Cardiology Section, Department of Medicine, University of Chicago, Chicago, IL, United States; ^4^Division of Cardiology, Department of Medicine, Loma Linda University Health, Loma Linda, CA, United States; ^5^Division of Diagnostic Imaging, Department of Diagnostic Radiology, University of Texas MD Anderson Cancer Center, Houston, TX, United States; ^6^Division of Cancer Medicine, Department of Lymphoma and Myeloma, University of Texas MD Anderson Cancer Center, Houston, TX, United States

**Keywords:** cardiac magnet resonance, CMR, diagnosis, prognosis, cardio-oncology

## Abstract

**Introduction:** Patients with systemic AL amyloidosis (AL) should be evaluated for cardiac amyloidosis (CA), as prognosis is strongly related to cardiac involvement. We assessed the characteristics of patients referred to cardiac magnetic resonance (CMR) with suspected CA from a cancer center and determine predictors of mortality/heart failure hospitalizations (HFH).

**Methods:** Forty-four consecutive patients referred for CMR with suspected CA were retrospectively included. Variables collected included cardiac biomarkers, in addition to echocardiographic and CMR variables. Survival analyses were performed to determine which variables were more predictive of mortality and HFH.

**Results:** Of the 44 patients included, 55% were females. 73% of patients were diagnosed with CA by CMR; 56% of them had an established diagnosis of AL. Patients with CA by CMR had higher native T1, higher extracellular volume (ECV) fraction, higher T2, less negative GLS by Echo, and higher troponin I and B-type natriuretic peptide (BNP). Kaplan-Meier survival analysis revealed that the following were predictive of mortality: an ECV ≥ 0.50 (*p* = 0.0098), CMR LVEF < 50% (*p* = 0.0010), T2/ECV ≤ 100 (*p* = 0.0001), and troponin I > 0.03 (*p* = 0.0025). In a stepwise conditional Cox logistic regression model, the only variable predictive of a composite of mortality and HFH was ECV (HR: 1.17, 95% CI = 1.02–1.34 *p* = 0.030).

**Conclusion:** ECV seems to be an important biomarker that could be a predictor of outcomes in cardiac AL amyloidosis. In combination, CMR and serum cardiac biomarkers might help to establish prognosis in patients with CA.

## Introduction

Patients with multiple myeloma (MM) or monoclonal gammopathy of undetermined significance (MGUS) are at an increased risk of developing AL amyloidosis (AL) (also referred to as primary systemic amyloidosis or primary amyloidosis) (1, 2). AL occurs due to abnormally functioning plasma cells that produce large amounts of the light-chain component of immunoglobulins. Typically, amyloid proteins are soluble in the plasma. However, these proteins may become insoluble after assembling into a misfolded “beta-sheet” conformation (3). Amyloidosis refers to the pathological accumulation of amyloid in the extracellular space of various organs (3, 4).

Amyloid can accumulate in the heart, which is referred to as cardiac amyloidosis (CA) (5–7) and can lead to a restrictive cardiomyopathy. CA can also lead to arrhythmias, heart blocks, or reduced QRS voltages (8, 9). Patients with AL should be evaluated for CA, as the prognosis of AL is greatly influenced by the presence or absence of cardiac involvement (10). In fact, one study demonstrated that cardiac involvement was the single most important determinant of prognosis in patients with evidence of systemic amyloidosis (11).

The gold standard for diagnosing CA is performing myocardial biopsy (3) and analyzing the sample using mass spectrometry (12). However, this procedure is invasive and may fail to detect amyloidosis if the sample is taken from a region without any amyloid deposition (3). Today, various serum biomarkers and imaging findings can assist physicians with the diagnosis and management of CA. Previously, echocardiography was frequently used to identify and prognosticate patient with CA (13–15). More recently, CMR has emerged as an important tool to diagnose and determine the prognosis of patients with CA (14, 16, 17). CMR has demonstrated to have great prognostic value in CA; in particular, T1 mapping and Extracellular volume fraction (ECV) have been validated to be predictive of mortality among patients with CA (18). T2 values have been found prognostic in AL CA (19). However, there is data that suggest that T2 times are no different from controls or not prognostic (20, 21). Thus, the association between native T2 times on CMR and prognosis in CA still remains unclear.

We assessed the characteristics of patients who underwent cardiac magnetic resonance (CMR) for suspicion of CA at a large tertiary cancer center in our pilot study. We also sought to determine which serum and imaging biomarkers were most predictive of heart failure hospitalizations (HFH) and mortality.

## Methods

After obtaining Institutional Review Board approval, we included 44 consecutive patients with suspected AL CA that underwent CMR in this retrospective observational study. Patients included in our cohort had a diagnosis of a hematological malignancy at risk for AL or a diagnosis of AL without a prior diagnosis of CA. They were evaluated by the myeloma department at a large tertiary cancer center, and they were referred for CMR with clinical suspicion of AL CA from March 1, 2009, to March 1, 2018. We retrospectively collected demographic information including age, gender, and body surface area (BSA). From the chart review, we collected past medical history information including the presence of any hematologic diagnosis (MM, MGUS, etc.), hypertension (HTN), diabetes (DM), hyperlipidemia (HLD), atrial fibrillation, stroke (CVA), and transient ischemic attack (TIA). We also recorded the presence of any episodes of ventricular tachycardia (VT), high-degree atrioventricular block, HFH, and survival. Next, we recorded the results of baseline serum tests including brain natriuretic peptide (BNP), troponin I, troponin T, blood urea nitrogen (BUN), creatinine (Cr), and hematocrit (Hct) (recorded nearest to the date of CMR).

### Echocardiography

Comprehensive echocardiographic examinations were performed using multiple commercially available equipment (GE Healthcare, Milwaukee, WI, USA; Philips, Amsterdam, The Netherlands) with 3.5-MHz ultrasound probes. Standard views were acquired carefully to avoid foreshortening. When feasible and clinically appropriate, we obtained live global longitudinal strain (GLS) measurements from four-, three-, and two-chamber apical long-axis views acquired at a frame rate of 50–70 frames per second by semiautomatic speckle tracking technique (EchoPAC, GE Medical Systems, Milwaukee, WI, USA).

We recorded echocardiographic information including left ventricular end diastolic volume (LVEDV), left ventricular end systolic volume (LVESV), left ventricular ejection fraction (LVEF), and GLS measurements (when available). Board-certified cardiologists reviewed and interpreted images and measurements.

### Cardiovascular Magnetic Resonance

All CMR images were acquired using a 1.5-T MRI scanner which was either Siemens Avanto (Siemens, Erlangen, Germany) or a 1.5-T GE AW (GE, Milwaukee, WI). A standard CMR exam consisted of the following: cine was performed for anatomical and functional assessment using a steady-state free-precession sequence with repetition time, 3.0 ms; echo time, 1.5 ms; in-plane spatial resolution, 1.7 to 2.0 ×1.4 to 1.6 mm; slice thickness, 8 mm; temporal resolution, 35–40 ms. Delayed enhancement (DE) was performed for tissue characterization using a segmented inversion-recovery sequence (12) (in-plane spatial resolution, 1.8 ×1.3 mm; slice thickness, 8 mm; temporal resolution, 160–200 ms) 10–15 min after intravenous contrast administration (gadopentetate dimeglumine, 0.125 mmol/kg). Cine- and DE-CMR images were obtained in matching short- and long-axis planes. Short-axis images were acquired every 1 cm (gap, 4 mm) throughout the entire LV. Long-axis images were obtained in standard two-, three-, and four-chamber orientations. For DE-CMR, inversion times were adjusted to null viable myocardium (13). Modified Look-Locker (MOLLI) T1 5(3)3 for long T1 (native T1) and MOLLI T1 4(1)3(1) for short T1 (post-contrast T1) were acquired in a mid-short-axis segment in patients scanned in Siemens Avanto. Pre-contrast T2 maps were obtained in the same locations as T1 maps using a FLASH sequence with T2 preparation pulses. From automated T1 and T2 maps, measurements were acquired. Native T1, T2, and post-contrast T1 were carefully measured in a global region of interest (ROI) at the mid-ventricular septum; meanwhile in native T1 and post-contrast T1, an ROI was drawn in blood pool to measure blood T1 times. No T1 and T2 mapping data was available from studies acquired in GE MRI scanners. ECV was calculated with the closest hematocrit value to the day of CMR acquisition. ECV was calculated using the following equation (18):

$$\begin{array}{l} ECV=\left(1-Hct\right)\;x\frac{\left[R1\;postcontrast\;myo-R1\;precontrast\;myo\right]}{\left[R1\;postcontrast\;blood-R1\;preconstrast\;blood\right]}\\ \;R1=\frac{1}{T1} \end{array}$$

A level 3 CMR cardiologist and a cardiac radiologist reviewed the CMR studies. The diagnostic impression from the LGE of each CMR was recorded (in particular, whether or not diagnostic for CA). Next, we recorded information on mortality (and date of death, when applicable) and number of HFH (and dates of admission, when applicable), in addition to the date of first and last office visit at our institution.

We collected CMR variables including left ventricular mass (LV mass), LVEDV, LVESV, LVEF, and pre-contrast and post-contrast native T1 times, respectively, in addition to pre-contrast native T2 times. We also utilized the native T1 times and hematocrit (the closest to the day of CMR) to estimate ECV.

We wanted to explore how T2 contributed to patients' morbidity and mortality. The notion of high T2 values in myocardium representing myocardial edema has a fair amount of bioplausibility in its relationship with mortality in some studies of CA. However, in some studies, it has not shown to be predictive. We evaluated the potential of T2/ECV for prognostication.

Kaplan–Meier and stepwise logistic regression analyses were performed to determine which variables were most predictive of mortality, HFH, and a composite of death and HFH. An event was cataloged as an HFH if during the day of admission the patient had a diagnosis of acute decompensated heart failure confirmed by a cardiologist's note. Group comparisons of CMR, echocardiography, and serum biomarkers between patients with CMR diagnosis of CA and patients without it, helped select the different cutoffs. IBM SPSS Statistics v.24 (IBM, Armonk, NY) and MedCalc 18.9 (MedCalc Software, Belgium) were used for statistical analysis. Significance was determined if *p* < 0.05.

## Results

Of the 44 patients included, 55% were females. Hematologic diagnoses at the time of CMR included 16 patients with MM, 20 patients with AL, seven patients with MM and concomitant AL, and 1 patient with lymphocytic lymphoma. 73% of patients were diagnosed with CA by CMR, and 56% of them had an established diagnosis of AL. Mean follow up was 434 days. These patients referred to CMR had at least one abnormal serum biomarker or at least one of the ventricular walls was thicker than 1.1 cm by echocardiogram at the parasternal long axis view.

Patients with CA by CMR had statistically significant higher troponin I and B-type natriuretic peptide (BNP), native T1, native T2, ECV, less negative Echo GLS, and lower T2/ECV ratio (see Table 1).

**Table 1:** Comparative table of patients with AL cardiac amyloidosis by CMR LGE criteria with patients without it.

**Variable**	***n***	**CMR with cardiac amyloidosis**	**CMR without cardiac amyloidosis**	**Mann-Whitney** ***p*-value**
Troponin I (ng/mL)	34	0.12 (0.01 to 1.05)	0.03 (0.01 to 0.03)	0.012
BNP (pg/mL)	35	794.40 (82.00 to 3830.00)	130.00 (19.00 to 396.00)	0.007
Echo GLS	30	−12.78 (−21.6 to −4.4)	−17.59 (−22.1 to −12.3)	0.037
Native T1 (ms)	30	1142.60(937.00 to 1251.00)	1057.30 (980.00 to 1144.00)	0.009
T2 (ms)	30	53.30 (41.00 to 60.00)	48.70 (44.00 to 53.00)	0.016
ECV	27	0.48 (0.27 to 0.88)	0.32 (0.22 to 0.52)	0.008
T2/ECV	27	121.42 (56.80 to 182.19)	164.73 (101.61 to 198.69)	0.017

**Figure 1:**
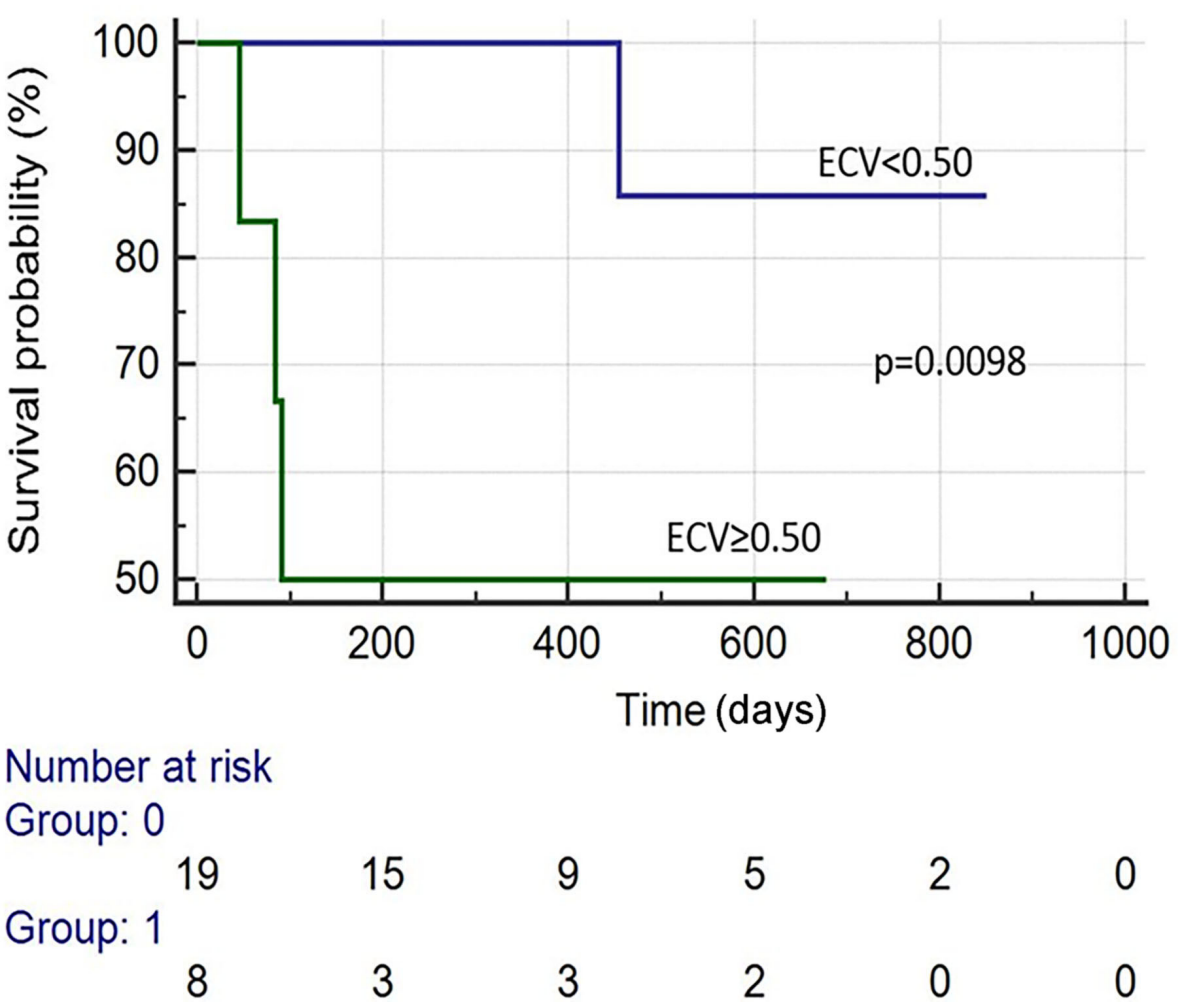
Survival curves of patients with suspected AL cardiac amyloidosis based on ECV by CMR.

**Figure 2:**
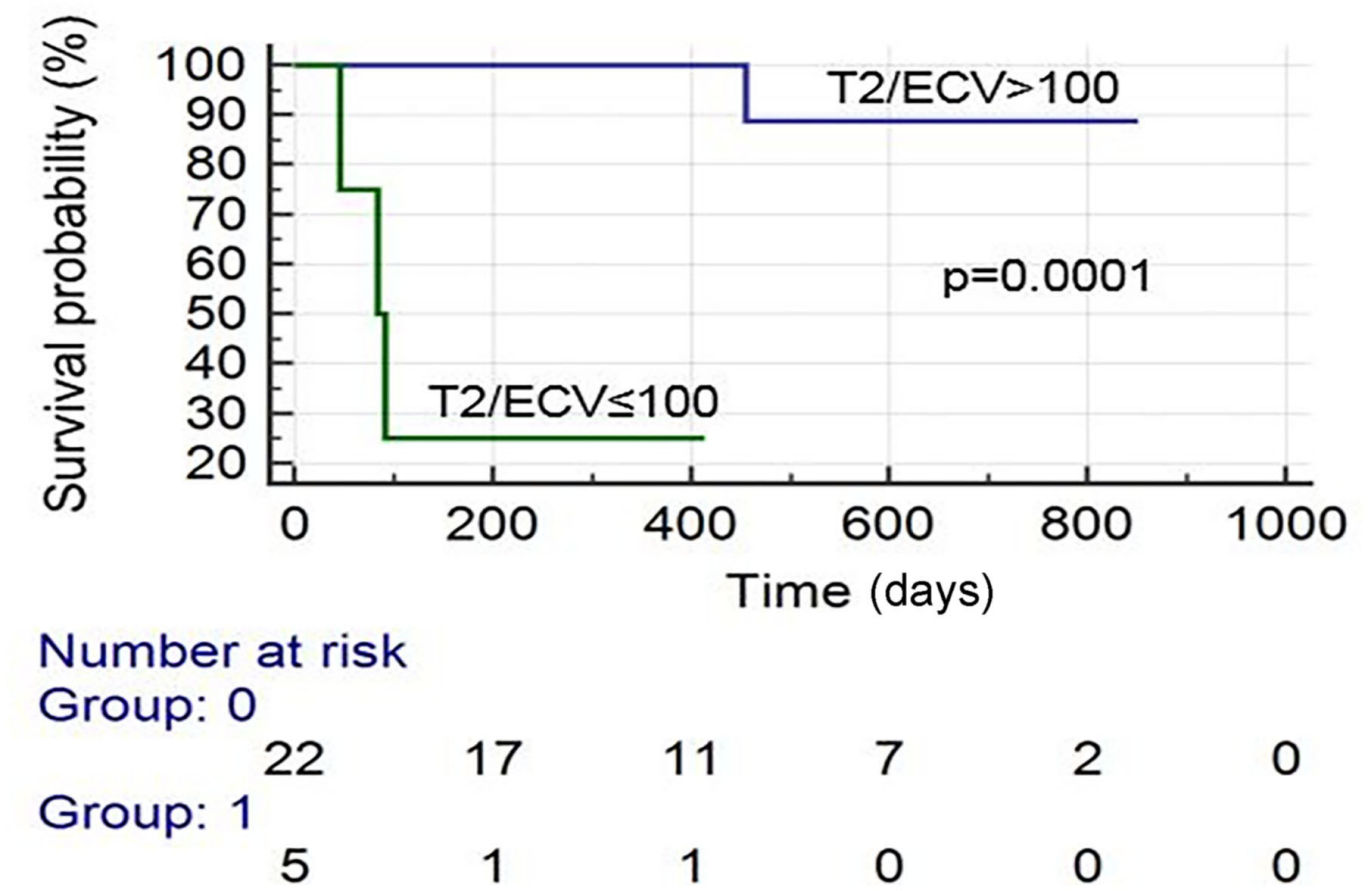
Survival curves of patients with suspected AL cardiac amyloidosis based on T2/ECV by CMR.

**Figure 3:**
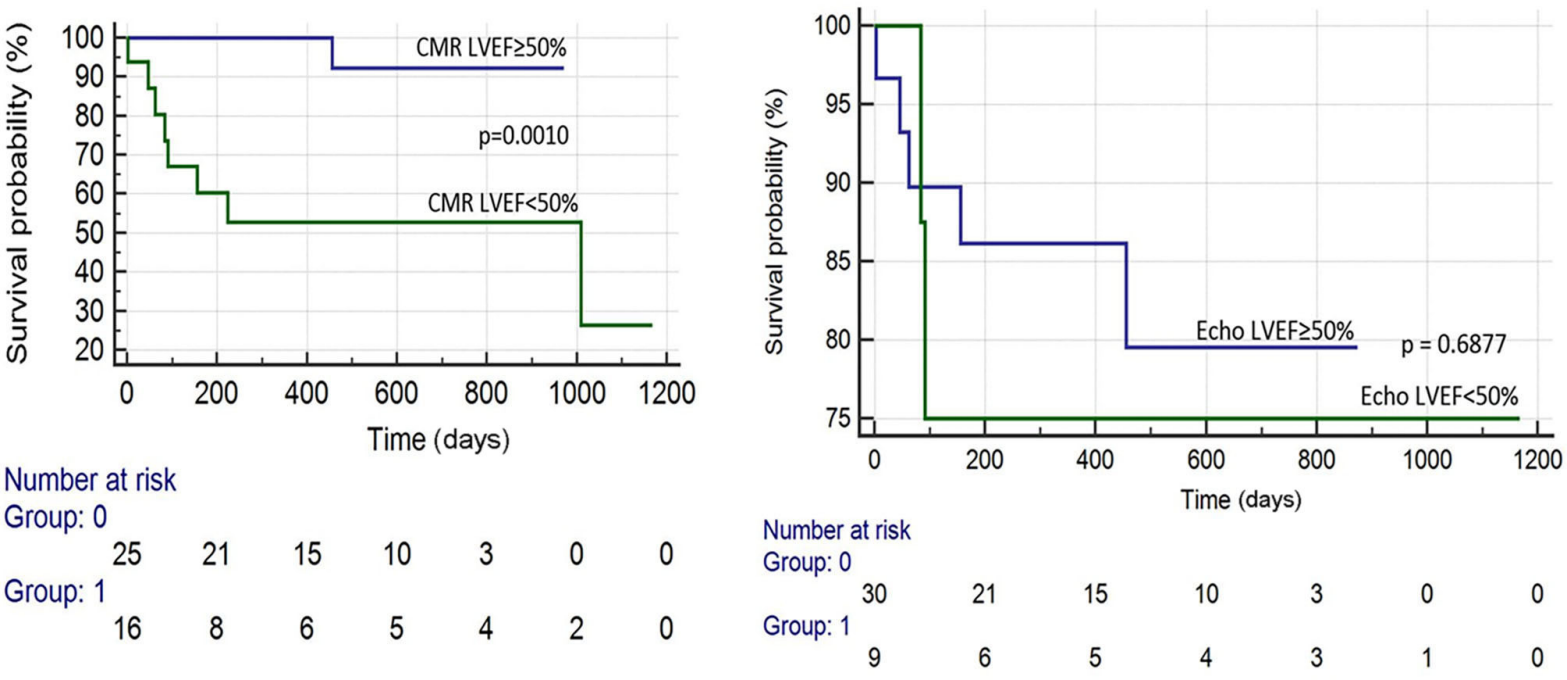
Survival curves by LVEF technique.

**Figure 4:**
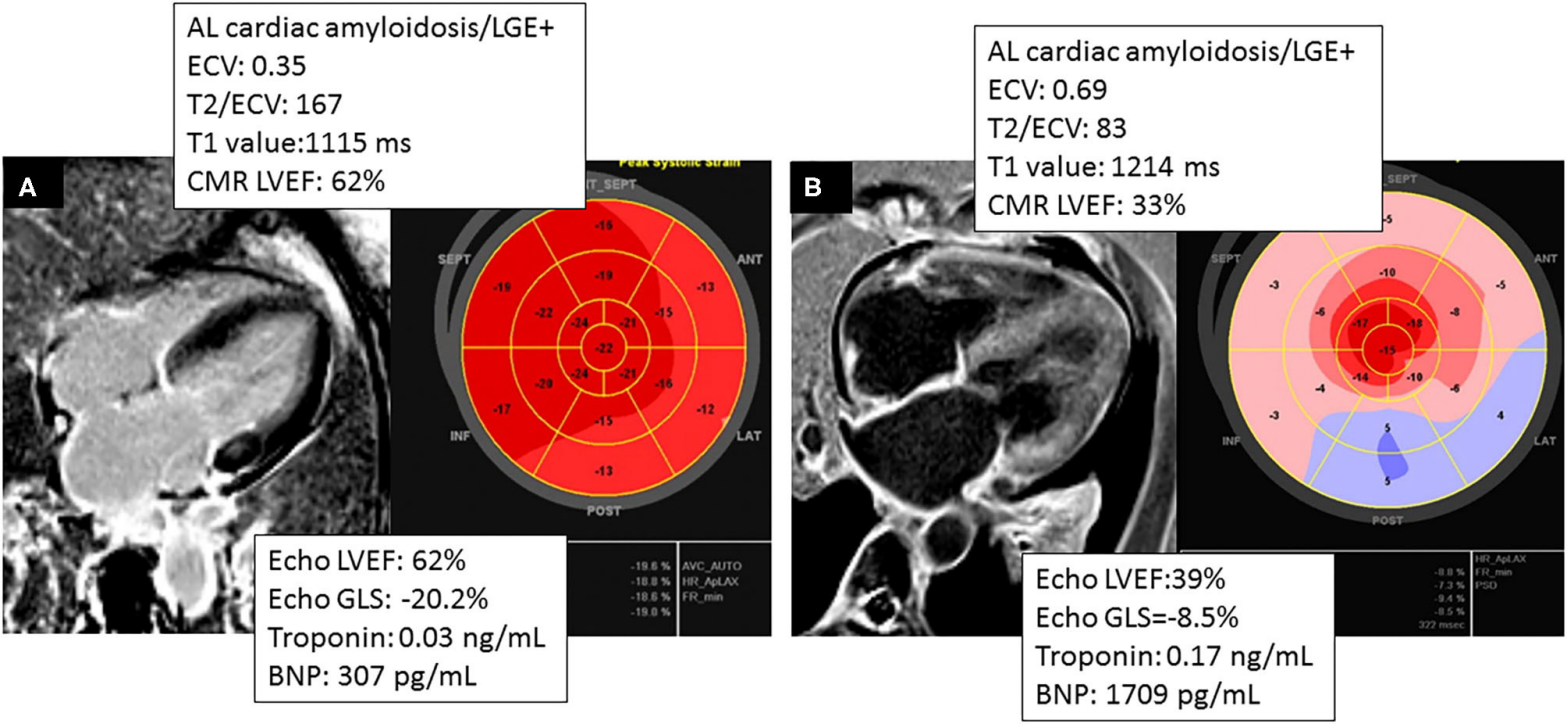
Comparing two different phenotypes of patients with AL cardiac amyloidosis. **(A)** CMR and TTE acquired within 2 months of a patient with AL cardiac amyloidosis with significant burden of disease by CMR (ECV = 0.35) and normal GLS by Echo and concordant LVEF in Echo and CMR. **(B)** Patient that had CMR and TTE acquired within 36 h, showing higher burden of disease by CMR (ECV = 0.69) and concordant LVEF in CMR and TTE.

There were 19 total events: 11 deaths and 8 HFH. Kaplan–Meier survival analysis revealed that the following were predictive of mortality: BNP > 300 pg/ml (*p* = 0.041), troponin I > 0.03 ng/ml (*p* = 0.002), an ECV ≥ 0.50 (*p* = 0.010), LVEF (CMR) <50% (*p* = 0.001), and T2/ECV ratio ≤ 100 (*p* < 0.001). The variables predictive of HFH were BNP > 300 pg/ml (*p* = 0.008), troponin I > 0.03 (*p* = 0.002), ECV ≥ 0.50 (*p* = 0.002), and T2/ECV ratio ≤ 100 (*p* < 0.001) (see Figures 1–4). T2 values by themselves were not significantly associated with mortality or HFH; neither were native T1, LVEF by echocardiography, or Echo GLS. In a stepwise conditional Cox logistic regression model including LVEF (CMR),Troponin I, T2/ECV, BNP, and ECV, the only one predictive of a composite of mortality and HFH was ECV (HR: 1.17, 95% CI = 1.02–1.34 *p* = 0.030).

## Discussion

ECV and T2/ECV were predictive imaging biomarkers, outperforming traditional serum biomarkers such as troponin I and BNP in this small cohort with low event rates. However, ECV was the most predictive of adverse events in a composite that included HFH and overall mortality per Cox logistic regression. Prior studies have demonstrated that serum cardiac biomarkers have prognostic value in CA (22, 23). In a study performed at the Mayo Clinic, AL amyloid patients with neither of these biomarkers elevated were considered stage I, patients with one of these biomarkers elevated were considered stage II, and patients with both of these biomarkers were considered stage III. The median survivals of these three groups were 26.4, 10.5, and 3.5 months, respectively (22, 23). Our findings were consistent with these results, as patients with CA diagnosed on CMR had elevated levels of troponin I and BNP. Furthermore, troponin I > 0.03 ng/ml was predictive of mortality. Echocardiogram has proven to be a useful tool for identifying and prognosticating CA. The most common feature of CA on echocardiogram is increased left ventricular wall thickness, often > 12 mm (9). Another common feature of CA on echocardiogram is the “speckled” pattern, which occurs because amyloid protein infiltrates are more echogenic than the surrounding myocardium (9). Left atrial enlargement, or either preserved or reduced systolic function (in the clinical setting of congestive heart failure), may also be noted on echocardiogram (24). With respect to GLS, CA demonstrates a typical “apical sparing” pattern (25). A decrease in GLS can be identified before a decrease in LVEF (26), suggesting that it may be a sensitive method for detecting myocardial dysfunction in CA. A GLS value equal or less negative than −14.81% has been demonstrated to predict mortality in patients with AL and a normal ejection fraction (EF) (27). Additionally, a GLS of −17% or more negative has been shown to predict survival among patients with AL amyloidosis undergoing autologous hematopoietic stem cell transplantation (28). Consistent with these findings, our study demonstrated that patients with CA on CMR have less negative GLS on echocardiogram. However, its performance when compared to ECV and T2/ECV was worst and less predictive in a smaller sample size.

A troponin I > 0.03 ng/mL, LVEF < 50% on CMR, and an ECV ≥ 0.50 on CMR were predictors of mortality. However, a T2/ECV ratio ≤ 100 was also associated with mortality, which has not been previously described in the literature. Further assessment of this ratio in larger studies is suggested. With respect to CMR, parametric imaging with T1 mapping has been shown to be a very useful tool with prognostic value in CA. Myocardial amyloid infiltration and fibrosis can lead to elevated non-contrast or native T1 relaxation times (29). A pre-contrast T1 time of >1,044 ms has been associated with a poor prognosis in AL amyloidosis (30). In our study, patients with CA on CMR had an elevated pre-contrast T1 time, but this was not predictive of mortality.

T1 mapping can also be used to estimate ECV, which can be used as a surrogate to *quantify* amyloid burden in myocardium (31). Previous studies have demonstrated that an ECV at equilibrium of >0.45 has been shown to portend a poor prognosis in AL amyloidosis (30). Likewise, we demonstrated that patients with CA on CMR had a higher ECV and that an ECV ≥ 0.50 was associated with increased mortality (see Figure 1).

The role of T2 mapping for the diagnosis and prognosis of CA has not been fully elucidated. One study assessed the mean T2 relaxation times of 49 patients with suspected CA who underwent CMR. There was no difference between the mean T2 relaxation times of those with biopsy-proven amyloidosis vs. those with negative biopsies (20). However, those patients with negative biopsies may have had another cardiomyopathy which may have led to elevated T2 times, or may have had amyloidosis not detected during biopsy (this is possible if an unaffected area of myocardium is biopsied). Our study reveals that native T2 times are indeed elevated among patients with AL CA on CMR, but values did not show prognostic capabilities. T2/ECV may be predictive of both mortality and HFH (see Figure 2). However, ECV was the most predictive variable by the Cox logistic regression model. We think that due to limitations in sample size and low event rate, T2/ECV was not a significant predictor by logistic regression and we recommend further studies to assess the potential of this ratio in predicting outcomes.

Interestingly, an LVEF <50% on CMR was predictive of mortality, whereas an LVEF <50% on echocardiography was not predictive of mortality (see Figure 3), suggesting that CMR LVEF measurements may have greater utility in determining prognosis among patients with CA (see Figure 4).

## Limitations

This study has limitations in sample size and selection bias of referring patients with clinical suspicion of CA. In our single center study, not all subjects underwent T1 and T2 mapping due to limitations in equipment. We acknowledge the limits of the predictive accuracy of our findings given the low event rate in our study. Because of the low event rate, multivariate analysis is limited. The number of subjects in the CMR-positive CA group (73%) far outnumbered the CMR-negative group for CA, which could have biased our results.

## Conclusion

ECV was the most predictive variable in this pilot study. We consider our findings as tentative. Our results were overall consistent with previous studies that demonstrated prognostic capabilities of cardiac biomarkers (troponin I and BNP) (32). GLS by speckle tracking echocardiography could establish a difference between presence and absence of AL CA by CMR (27) but failed to prognosticate mortality and HFH in our cohort. CMR findings of ECV (30) and T2/ECV prognosticated well in this study, and further studies with larger sample size warranted to assess better ECV and T2/ECV ability to prognosticate in AL CA given our small sample size and low event rate. Our study demonstrates that native T2 times are indeed elevated in AL CA, without effects in prognosis. CMR parametric measurements outperformed echocardiographic measurements such as GLS and LVEF in predicting both mortality and HFH. This study supports the importance of CMR in addition to serum cardiac biomarkers in predicting outcomes among patients suspected or at risk of having AL CA.

## Data Availability Statement

The original contributions presented in the study are included in the article/supplementary material, further inquiries can be directed to the corresponding author/s.

## Ethics Statement

The studies involving human participants were reviewed and approved by MD Anderson IRB, who provided exemption due to restrospective review. Written informed consent for participation was not required for this study in accordance with the national legislation and the institutional requirements.

## Author Contributions

AA wrote the manuscript. JL-M wrote and planned the manuscript. All the other authors edited the manuscript.

## Conflict of Interest

The authors declare that the research was conducted in the absence of any commercial or financial relationships that could be construed as a potential conflict of interest.
